# 

**Published:** 1895-06-15

**Authors:** 


JUNE 15TH, 1895.

PRICE TWOPENCE.

No. 455, Vol. XVIII,—June 15th, 1895.

[Registered at the General Post Office as a Newspaper.

WITH EXTRA NURSING SUPPLEMEN

Per Annum, 10s. 6d., post free.

Monthly Parts, 6d.; post free, 8d.

Ditto per Annum, 8s. 6d., post free.

### THE HOSPITAL

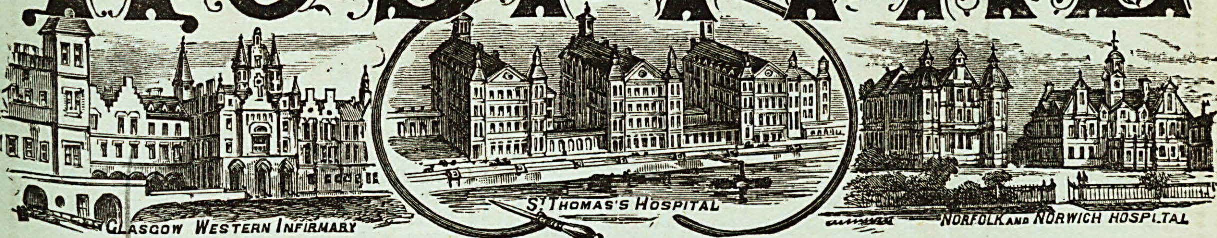

No. 455. Vol. XVIII. WITH EXTRA NURSING SUPPLEMENT. SATURDAY, JUNE 15TH, 1895.

### SPECIAL HOSPITAL SUNDAY NUMBER.

**WITH ILLUSTRATED SUPPLEMENT.**

